# Screening Colonoscopy Unmasking Colonic Metastasis from an Occult Breast Ductal Carcinoma: A Case Report and Review of the Literature

**DOI:** 10.1155/2019/8432079

**Published:** 2019-02-24

**Authors:** Bachar Samra, Sassine Ghanem, Ghulam Ilyas, Evelyn Taiwo

**Affiliations:** ^1^Department of Hematology/Oncology, State University of New York, Downstate, Brooklyn, NY, USA; ^2^Department of Pathology, State University of New York, Downstate, Brooklyn, NY, USA; ^3^Department of Hematology/Oncology, Kings County Hospital, Brooklyn, NY, USA

## Abstract

Metastatic spread from breast cancer to the gastrointestinal tract is rare. Such cases are predominantly lobular carcinomas and they usually occur later on during the course of disease progression with the stomach being the most common site involved. Furthermore, occult breast primary tumor is extremely uncommon. To the best of our knowledge, we describe here the first case of incidental colonic metastasis as first presentation of an occult breast ductal carcinoma. We also provide a review of the literature on gastrointestinal—and specifically colonic—involvement from breast ductal carcinoma.

## 1. Introduction

Breast cancer is the most common malignancy and second leading cause of cancer-related death in women with the predominant histology being invasive ductal carcinoma [1]. Usual sites of metastases (mets) include the lymph nodes, liver, lungs, bones, and brain. However, metastatic involvement of the gastrointestinal (GI) tract is extremely rare [2] and is predominantly of lobular histology [3]. The majority of reported cases of GI mets from breast cancers occur mainly upon disease progression or recurrence [4, 5] and rarely upon presentation. Furthermore, radiologically occult breast cancer (typically presenting as isolated axillary disease) is an uncommon entity accounting for <1% of all breast cancers [6]. We herein report the case of a postmenopausal woman who was found to have a metastatic colonic lesion on her screening colonoscopy along with asymptomatic diffuse bone metastases from an occult breast ductal carcinoma.

## 2. Case Presentation

**Figure 1:**
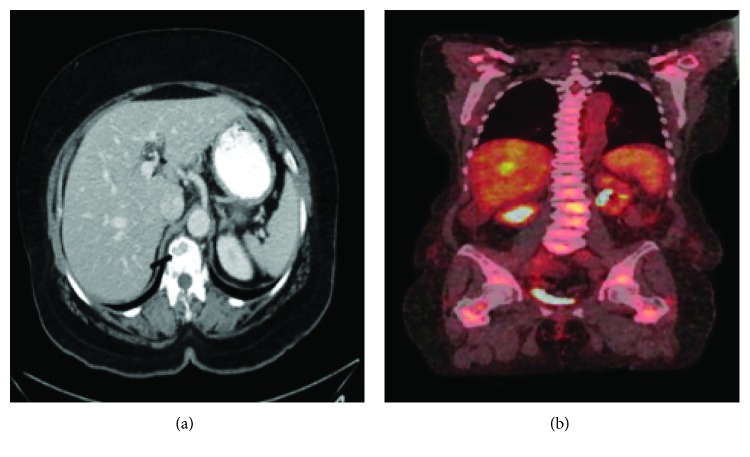
(a) Computerized-axial tomography showing multiple lytic vertebral metastases (arrow). (b) Positron Emission Tomography showing increased metabolic activity in the vertebral bodies and pelvis.

A 64-year-old postmenopausal and previously healthy woman was referred to our hospital in June 2015 after her first screening colonoscopy revealed an adenocarcinoma. Immunohistochemistry (IHC) of the colonic lesion was positive for CK-7, GATA-3, and ER, weakly positive for MOC-31, and negative for CK-20, CD-X2, PR, PAX-8, SOX-10, CD45, chromogranin, synaptophysin, and TTF-1, findings consistent with a breast primary. The patient was asymptomatic with no palpable masses or lymphadenopathy on clinical exam. Her past surgical history was significant for hysterectomy secondary to symptomatic fibroids, and her family history was negative for cancers. A screening mammography performed a month prior was normal. Staging with computerized tomography (CT) of the chest, abdomen, and pelvis in August of 2015 revealed osteolytic thoracic vertebral and pelvic lesions as well as bilateral axillary adenopathy measuring up to 1.4 cm on the right and 1.1 cm on the left. A Positron Emission Tomography (PET) CT was done in October of 2015 and revealed metabolic activity in the right axilla as well as patchy radiotracer uptake involving the vertebral column corresponding to the CT findings (Figure 1).

**Figure 2:**
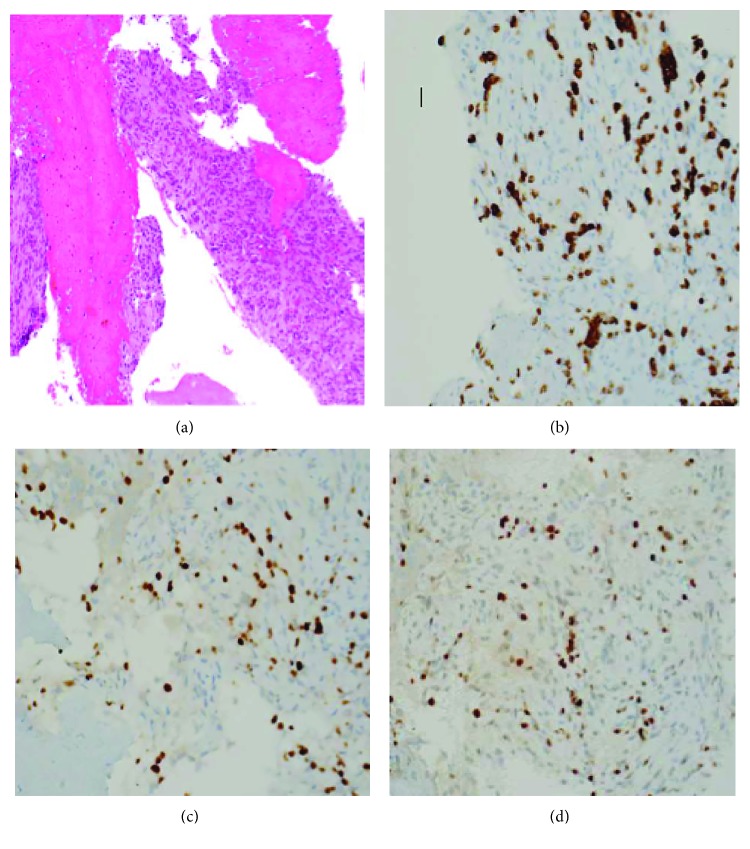
(a) Hematoxylin and eosin stain × 200x showing fragments of bone and the bone marrow is replaced by malignant spindle and epitheloid cells. Immunohistochemical stains, 400x: (b) CK7 3, (c) GATA3, and (d) ER all positive.

A fine needle aspiration (FNA) of the right axillary lymph node was performed in November of 2015 but was negative for malignancy. Of note, the CEA, CA 19-9, and CA-125 levels were all within normal limits (2.08 ng/mL, <1 U/mL, and 6.4 U/mL, respectively). The patient was not seen by oncologist until March of 2016 when she was started on anastrozole until further work up was done. A repeat mammography and breast ultrasound showed only bilateral axillary lymphadenopathy. A bone biopsy of a right sacral lesion confirmed metastatic breast invasive ductal carcinoma with IHC positive for CK-7, GATA-3, ER, and PR but negative for CD45, CD138, and HER2 (Figure 2).

Three months later, she was switched to palbociclib and letrozole in the hope of achieving longer disease control. She enjoyed stable disease for 18 months but eventually progressed in February 2018, presenting with weight loss, new lung, liver, and significant peritoneal carcinomatosis with ascites. Due to her rapid, aggressive, and symptomatic progression, she was initiated on chemotherapy with weekly paclitaxel 80 mg/m^2^, of which she only received 2 cycles due to neutropenia, worsening ascites, and lower extremity edema requiring hospitalization. Due to her intolerance of chemotherapy, she was switched to fulvestrant; however, she had no response and she required another hospitalization due to her symptomatic anasarca. After further discussion, the patient opted for hospice care and she unfortunately expired in April 2018 within a few days of hospitalization.

## 3. Discussion

The incidence of GI mets from breast cancer is rare, and most of the literature in this setting is derived from case reports and case series. In some autopsy series, the incidence has varied from <1% to 12% [2, 7, 8]. Such cases are predominantly hormone-receptor positive [9], and the stomach is the most common site involved regardless of histology. A literature review done by Ambroggi et al. revealed 206 cases of GI mets from breast cancer between 1943 and 2012, with the following organ distribution: stomach (60%), esophagus (12%), colon or small intestine (11%), rectum (7%), and oropharynx or anus (1%) [2]. Histopathologically, invasive ductal carcinoma (IDC), which accounts for more than 90% of all breast cancers, constitutes only 15-35% of breast cancers that are metastatic to the GI tract [3, 7]. It is well-known that IDC and invasive lobular breast carcinoma (ILC) have different metastatic behavior. ILC has a greater propensity to metastasize to the GI tract, gynecological organs, peritoneum, retroperitoneum, adrenal glands, and bone marrow, whereas IDC frequently metastasizes to the lungs, bones, liver, and brain [3, 10]. In the largest autopsy series (encompassing 2605 cases) that analyzed the differences in metastatic patterns between both different histological subtypes, the incidence of GI mets was 4.5% in LIC vs. 0.2% in IDC [3]. The reasons for such disparity are grossly unknown, but it has been suggested that the distinct ILC transcriptomic signature [11] and the absence of the cell-cell adhesion molecule E-CAD, which can decrease the adhesiveness of tumor cells, may explain the unique metastatic patterns of ILC [12]. It is also worth noting that ILC tends to metastasize in a more “diffuse” pattern such as linitis plastica in the stomach, with peritoneal mets and signet cell appearance, as opposed to the nodular appearance seen in IDC [13].

Therefore, colonic metastasis from breast IDC is extremely rare. After reviewing the literature and excluding the cases of ILC, metaplastic, mixed, and unknown histology, we identified only 21 published cases of breast IDC with colonic mets, 2 of them were males (see Table 1).

Among those cases, the median age was 58 years (range 43-76 years). The median time from breast cancer diagnosis to GI involvement was 4.5 years (range 1 year-28 years). However, in our patient, the GI involvement was the first manifestation of her metastatic disease, making our case more unique. Since our patient was asymptomatic at diagnosis, and her colonic lesion was detected on screening colonoscopy and not on standard imaging including PET/CT, this raises the question whether the general incidence of colonic or GI mets from breast cancer may in fact be underrecognized at diagnosis without endoscopic investigation. However, our patient became significantly symptomatic upon progression from her peritoneal carcinomatosis and ascites.

Interestingly, our patient also had no primary breast tumor on physical exam, ultrasound, and mammography. Occult breast cancer by mammography and ultrasound is uncommon, with an estimated incidence of less than 1% of all breast cancers. Of note, a SEER database analysis showed better survival for occult breast cancers compared with nonoccult breast cancers after adjusting for clinicopathological features [6]. Due to higher sensitivity compared with mammography, MRI can detect up to 25% of otherwise occult breast tumors [32]. However, this difference in tumor detection is only significant in the case of fibroglandular or dense breasts, which are typically seen in patients that are younger than 50 [33], as well as multifocal-multicentric disease and tumors that are less than 5 mm in size [34]. Nonetheless, despite advances in radiological imaging techniques and the use of high sensitivity MRI in the modern era, which make the diagnosis of occult disease more accurate, few recent case series reported that radical mastectomy could fail to identify a primary tumor in more than 10% of radiologically occult breast cancers [35, 36]. These findings suggest that occult disease may be a true histological absence of tumor in the breast tissue and not a matter of under detection by radiological tests. It has also been hypothesized that this may be due to a primary axillary tumor originating from heterotopic mammary tissue in the axilla [37, 38]. However, because our patient was 64 years old with fatty pattern breasts, the likelihood of detecting a primary breast tumor by MRI after a negative mammogram/ultrasound is likely to be low. Furthermore, in the presence of metastatic disease and the lack of indication for locoregional therapy, the need for a breast MRI was abrogated in our case. Since our patient only had an FNA of her right axillary lymph node, which was negative for both cancer and glandular tissue, the possibility of an ectopic axillary breast tissue with carcinoma is not ruled out. In even extremely more rare cases, GI involvement can be the first manifestation of occult metastatic breast carcinoma as illustrated in 3 published cases, which were all LICs (with stomach [39], gastric linitis [40], and pancreatic involvement [41]). However, we have not identified a similar case with IDC, which makes our patient the first case presenting with colonic mets from an occult breast ductal carcinoma.

The clinical presentation of colonic mets from breast cancer can mimic a primary colon adenocarcinoma as symptoms can be nonspecific, albeit nonexistent, and radiological/endoscopic findings can be similar. Pathologically, the absence of transition between GI mucosa and tumor favors a metastatic growth. By IHC, breast carcinoma is typically CK7, GATA3 positive, and CK20 negative, whereas colon carcinoma is CK20 and CD-X2 positive but CK7 negative.

The rarity of GI or colonic mets from breast carcinomas precludes the realization of prospective clinical trials dedicated to this population. Given the scarcity of clinical data and guidelines specific to GI mets, physicians are left with using the appropriate treatment options that are commonly used for metastatic breast carcinoma in general. In our literature review of GI mets from breast cancer, the majority of cases were treated with chemotherapy despite being hormone-receptor positive, HER2 negative. However, such cases were significantly symptomatic with rapid progression or recurrence unlike our patient who was asymptomatic from her colonic and bone mets. Surgery and radiation therapy are usually reserved for local palliative indications such as bleeding or obstruction, which were not present in our case. According to McLemore et al., surgical intervention did not have a significant effect on survival (28 vs. 26 months, *p* = .31). Patients with metastasis only to the GI tract who underwent palliative surgical resection had numerically longer median survival (44 vs. 9 months, *p* = .1) [7]; however, this difference was not statistically significant. Our patient was treated according to the standard of care by combining endocrine therapy (aromatase inhibitor) with a cyclin-dependent kinase 4/6 inhibitor (palbociclib) [42]. Upon progression afterwards, her disease was significantly symptomatic and rapidly growing warranting the use of standard chemotherapy with single-agent paclitaxel, which was switched later due to intolerance to fulvestrant in concordance with the current guidelines. In terms of prognosis, it is unclear whether GI or colonic mets independently confer a poorer prognosis in breast cancer. The overall survival in the reported cases has ranged from few months (especially in the setting of bleeding/obstruction) up to 5 years [25]. In our literature review of other breast IDC with colonic mets, the median overall survival was 12 months (range 6-60 months); however, this has many limitations. Ten of the published cases (47%) did not report on survival, and most of the cases with reported survival were still alive at the time of publication. Furthermore, the nature of this retrospective series with many old case reports and many confounding factors makes the generalization of such outcome highly discouraged, especially in the modern era where our therapeutic arsenal for metastatic breast cancer has significantly improved. Our patient survived 35 months from the time of diagnosis including 26 months since treatment initiation. The fact that she remained relatively asymptomatic for about 9 months before starting treatment, while taking into consideration the lead time of bias of a screening colonoscopy, reflects a favorable tumor biology at presentation.

## 4. Conclusion

In conclusion, gastrointestinal metastases from breast cancer are rare. Most cases are of lobular histology, hormone receptor positive, and they typically present years after diagnosis, with the stomach being the predominant site. On the other hand, radiologically occult breast cancer is an extremely rare entity. To our knowledge, our patient is the first reported case of an occult invasive breast ductal carcinoma with colonic metastases as initial presentation. This should raise awareness among oncologists about gastrointestinal involvement in breast cancer including ductal carcinomas in order to make early and accurate diagnosis and appropriate management.

## Figures and Tables

**Table 1:** Case reports of breast invasive ductal carcinomas with colonic metastases.

Reference	Age/sex	Receptor status	Other metastatic sites involved	Time from diagnosis	Treatment	Survival
Samo et al. [14]	76/f	ER+	Bone	4 years	Ileocecectomy with adjuvant chemotherapy cyclophosphamide and doxorubicin	NR
Shimonov and Rubin [15]	65/f	NR	None	2 years	Sigmoidectomy, post-op cisplatin, Adriamycin, and 5-fluorouracil x6	Alive at 46 months
	72/f	NR	None	6 years	Sigmoidectomy, not a candidate for adjuvant therapy	Died at 42 months
Koutsomanis et al. [16]	65/f	NR	None	4 years	Partial colectomy, post-op Endoxan, epirubicin, and 5-fluororacil	Alive at 36 months
Vaidya et al. [17]	56/f	ER-, PR-	None	5 years	Partial colectomy and post-op chemotherapy	NR
Uygun et al. [18]	43/f	ER-, PR+	Bone	3.5 years	Right hemicolectomy then 5-fluorouracil, Adriamycin, and cyclophosphamide x6	Alive at 7 months
Théraux et al. [19]	69/f	ER+, PR+, HER2-	Brain and lung	28 years	Partial colectomy and anastrozole	Alive at 12 months
Feng et al. [20]	49/f	NR	NR	2 years	NR	NR
Birla et al. [21]	72/f	ER-, PR equivocal, HER2-	None	3 years	Partial colectomy, did not tolerate chemotherapy	NR
Koleilat et al. [4]	54/m	ER+, PR+, HER2-	Chest wall	14 years	Subtotal colectomy then Taxotere and Xeloda	Alive at 10 months
Rajan et al. [22]	60/f	ER+, PR-, HER2-	Bone	21 years	Preoperative radiation then partial colectomy	NR
Hsieh et al. [5]	50/f	ER+, PR+, HER2+	None	16 years	Right hemicolectomy and ileotransversotomy followed by hormone therapy	Alive at 6 months
Horimoto et al. [23]	47/f	ER+, PR-, HER2-	None	5 years	Aromatase inhibitor	Alive at 12 months
Zhou et al. [24]	54/f	ER-, PR-, HER2-	None	9 years	Second line chemotherapy and endocrine Therapy	Alive at 12 months
Schellenberg et al. [25]	69/f	ER+, PR-, HER2-	Peritoneal carcinomatosis	2 years	Unclear	Died after 60 months
Katz et al. [26]	68/f	ER+, PR+, HER2+	Bone metastasis	15 years	Switched from letrozole to exemestane	NR
Michalopoulos et al. [27]	55/f	NR	None	4 years	Right hemicolectomy for stricture with postop chemotherapy	Alive at 36 months
Yokota et al. [28]	57/f	NR	Peritoneal carcinomatosis/ascites	10 years	Total colectomy for ascending colon stricture and peritoneal implant	NR
Taal et al. [29]	NR	NR	NR	NR	NR	NR
Jones et al. [30]	55/m	ER+, PR+, HER2-	Mediastinal and axillary lymph nodes	4 years	Switched from tamoxifen to anastrozole	NR
Murukutla et al. [31]	59/f	ER+, PR+, HER2-	None	1 year	NR	NR

F: female; M: male; NR: not reported; ER: estrogen receptor; PR: progesterone receptor; HER2: human receptor growth factor receptor.
